# Modeling immune functions of the mouse blood–cerebrospinal fluid barrier in vitro: primary rather than immortalized mouse choroid plexus epithelial cells are suited to study immune cell migration across this brain barrier

**DOI:** 10.1186/s12987-016-0027-0

**Published:** 2016-01-29

**Authors:** Ivana Lazarevic, Britta Engelhardt

**Affiliations:** Theodor Kocher Institute, University of Bern, Freiestrasse 1, 3012 Bern, Switzerland

**Keywords:** Blood cerebrospinal fluid barrier, Choroid plexus, Immortomouse^®^, ECPC4

## Abstract

**Background:**

The blood–cerebrospinal fluid barrier (BCSFB) established by the choroid plexus (CP) epithelium has been recognized as a potential entry site of immune cells into the central nervous system during immunosurveillance and neuroinflammation. The location of the choroid plexus impedes in vivo analysis of immune cell trafficking across the BCSFB. Thus, research on cellular and molecular mechanisms of immune cell migration across the BCSFB is largely limited to in vitro models. In addition to forming contact-inhibited epithelial monolayers that express adhesion molecules, the optimal in vitro model must establish a tight permeability barrier as this influences immune cell diapedesis.

**Methods:**

We compared cell line models of the mouse BCSFB derived from the Immortomouse^®^ and the ECPC4 line to primary mouse choroid plexus epithelial cell (pmCPEC) cultures for their ability to establish differentiated and tight in vitro models of the BCSFB.

**Results:**

We found that inducible cell line models established from the Immortomouse^®^ or the ECPC4 tumor cell line did not express characteristic epithelial proteins such as cytokeratin and E-cadherin and failed to reproducibly establish contact-inhibited epithelial monolayers that formed a tight permeability barrier. In contrast, cultures of highly-purified pmCPECs expressed cytokeratin and displayed mature BCSFB characteristic junctional complexes as visualized by the junctional localization of E-cadherin, β-catenin and claudins-1, -2, -3 and -11. pmCPECs formed a tight barrier with low permeability and high electrical resistance. When grown in inverted filter cultures, pmCPECs were suitable to study T cell migration from the basolateral to the apical side of the BCSFB, thus correctly modelling in vivo migration of immune cells from the blood to the CSF.

**Conclusions:**

Our study excludes inducible and tumor cell line mouse models as suitable to study immune functions of the BCSFB in vitro. Rather, we introduce here an in vitro inverted filter model of the primary mouse BCSFB suited to study the cellular and molecular mechanisms mediating immune cell migration across the BCSFB during immunosurveillance and neuroinflammation.

## Background

The choroid plexus (CP) is a highly-vascularized structure that folds from the ependymal lining of the lateral, third and fourth ventricles in a convoluted fashion into cerebrospinal fluid (CSF)-filled ventricular spaces. In contrast to brain microvessels at the blood–brain barrier (BBB), CP microvessels are fenestrated and allow for free diffusion of molecules from the blood into the CP stroma. Highly-polarized epithelial cells, which cover the entire surface of the CP, establish a barrier between the blood and the CSF, the blood–cerebrospinal fluid barrier (BCSFB). While preventing the free diffusion of blood components into CSF, the BCSFB regulates the transport of metabolites and is considered the major source of the CSF (reviewed in [1, 2]). Paracellular diffusion of molecules is prevented by unique tight junctions (TJs) constituted by the oligodendrocyte specific protein (OSP/claudin-11) that runs in parallel strands around the entire circumference of the CP epithelial cells [2, 3]. In addition to claudin-11, BCSFB TJs are composed of claudin-1, -2 and -3, occludin, and junctional adhesion molecules A and B (JAM-A and JAM-B), while the zonula occludens-1, 2, 3 (ZO1, 2, 3) scaffolding proteins connect BCSFB TJ proteins to the actin cytoskeleton (summarized in [4]). The adherens junctions (AJ) of the BCSFB are formed by homophilic interactions of the transmembrane epithelial cadherin (E-cadherin) that is linked to the actin cytoskeleton via α- and β-catenin [5, 6].

Over the last decades, evidence has accumulated for a potential role of the choroid plexus, and thus specifically the BCSFB, in controlling immune cell entry into the CNS. It has been shown that during experimental autoimmune encephalomyelitis (EAE), an animal model for multiple sclerosis (MS), functional expression of intercellular adhesion molecule (ICAM)-1 and vascular cell adhesion molecule (VCAM)-1 is upregulated on choroid plexus epithelial cells [3, 7, 8]. ICAM-1 and VCAM-1 mediate the migration of circulating autoaggressive T cells across the endothelial BBB in EAE (summarized in [9]) and have been suggested to also play a role in mediating immune cell trafficking across the BCSFB [10]. In addition, the recent observations that CCR6-deficient mice are resistant to EAE and that CCL20 is expressed by CP epithelial cells but not by the endothelial cells forming the BBB [11], support a role for the chemokine receptor CCR6 and its ligand CCL20 in mediating the migration of neuroantigen-specific CD4^+^ Th17 cells into the CNS. Last but not least, the CP mediates neutrophil invasion into the CNS after traumatic brain injury [12] and in bacterial and viral infections [13].

Beyond these observations, our knowledge on the cellular and molecular mechanisms involved in immune cell migration across the BCSFB during immunosurveillance and neuroinflammation is sparse. Live cell imaging of single immune cells crossing the BCSFB in vivo is hindered by the poor accessibility of the CP within the brain ventricular spaces and its constant movement due to the CSF pulsations. Thus, reliable in vitro models of the BCSFB that preserve the differentiated characteristics of the choroid plexus epithelium are mandatory to study immune cell migration across the BCSFB. Primary cultures of bovine, porcine, sheep, rabbit and rat choroid plexus epithelial cells have been established and proven as reliable in vitro models of the BCSFB of the respective species [14–20]. In addition, a number of cell lines, notably the rat cell line Z310 [21–24] and human cell lines originating from either CP papillomas [25, 26] or carcinomas [27], have been used to model the BCSFB in vitro. A number of laboratories have previously established in vitro cultures of primary mouse CP epithelial cells [28–31]. However, there is a lack of a comprehensive characterization of these models regarding purity and growth as contact-inhibited monolayers, expression of CP epithelial cell markers, maturation of TJs and functional barrier characteristics [30]. Furthermore, an optimization of these in vitro models, which would allow for the study of immune functions of the mouse BCSFB including immune cell migration into the CNS, is still missing [8]. Given the availability of gene-targeted mice, an in vitro model of the mouse BCSFB would be advantageous to define the role of individual molecules for BCSFB immune functions.

The aim of the present study therefore, was to establish in vitro models of the mouse BCSFB specifically suited to study immune cell migration across the BCSFB in a two-chamber setup. To this end we first established the isolation and primary culture of mouse CP epithelial cells from different mouse strains. This included the Immortomouse^®^, which expresses the temperature sensitive Simian Virus 40 (tsSV40) large T Antigen under the MHC class I promoter and has proven a successful source for establishing conditionally immortalized epithelial cell lines from the intestine and other tissues [32, 33]. In addition, we analyzed the ECPC4 cell line, which was produced from CP carcinomas of transgenic mice expressing the SV40 large T antigen [34].

The three in vitro BCSFB models were analyzed for the development of epithelial contact-inhibited monolayers, expression of cytokeratin and transthyretin as markers for epithelial cell maturation, maturation of BCSFB specific junctional complexes, and the establishment of a tight barrier with low permeability to small molecular tracers with a high transepithelial electrical resistance (TEER). Our novel Immortomouse^®^ derived lines and the ECPC4 cells did not fulfill these criteria as they lacked expression of cytokeratin and failed to grow into confluent monolayers with barrier characteristics. In contrast, highly-purified primary mouse choroid plexus epithelial cell (pmCPEC) cultures showed proper expression and distribution of CP epithelial markers including the junctional proteins and readily established a tight barrier. Inverted cultures, with pmCPECs grown on the lower side of a porous filter membrane, were found to be suitable in vitro models of the BCSFB to study the migration of encephalitogenic T cells across the BCSFB from the basolateral (blood facing) to the apical (CSF facing) side, which correctly models the in vivo situation.

## Methods

### Harvesting of choroid plexus tissues

Mice were sacrificed by CO_2_ inhalation according to the approved animal protocol (approval number BE72/15 of the Cantonal Veterinary Office of the Canton Bern). Organ harvest was thus conducted strictly in compliance with the legal and ethical requirements demanded by the national guidelines and the legislations for animal experimentations. This implements Directive 2010/63/EU on the protection of animals used for scientific purposes and thus incorporates the principle of the Three Rs.

### Primary mouse choroid plexus epithelial cell (pmCPECs) isolation and culture

Mouse choroid plexus epithelial cells (CPECs) were isolated by a modification of a protocol previously established for the isolation of rat CPECs [20]. In brief, sex-matched 6–10 week old C57BL/6 mice or Immortomice^®^ (see below) were euthanized with CO_2_, decapitated and the brain excised. For one preparation, CP was removed from 15 to 20 mice from the lateral and the 4th ventricles using a stereomicroscope. The CPs were collected, rinsed in Dulbecco’s phosphate buffered saline (DPBS, Gibco, Paisley, UK) and digested with 0.1 mg/ml pronase (Roche, Mannheim, Germany) in DPBS for 30 min at 37 °C in a shaking water-bath. The digestion was ended by addition of 5 ml DPBS without pronase. The CP clumps were re-suspended and recovered by sedimentation controlled visually by eye. The supernatant, which contained mostly single non-epithelial cells and debris, was discarded. The sedimented CP clumps were further mechanically and enzymatically disaggregated by pipetting the clumps up and down for 10 to 20 s (depending on the size of the clumps) in 1 ml of 0.025 % Trypsin–EDTA (1×) (Gibco, Paisley, UK) containing 12.5 μg/ml DNAse I (Roche, Mannheim, Germany). This procedure was followed by a sedimentation step at RT for 1–2 min and ended based on visual control of the sedimentation. The supernatant, which is enriched for single CP epithelial cells and thus more turbid, was transferred to a new tube containing 5 ml of CPEC medium (DMEM/F12 1:1 (Gibco, Paisley, UK), FBS 10 % (Gibco, Paisley, UK), 2 mM glutamine (Gibco, Paisley, UK), 50 μg/ml gentamycine (Gibco, Paisley, UK). The procedure of mechanical/enzymatic disaggregation and sedimentation was repeated 5× as this was found to yield the highest number of CP epithelial cells. The single cell suspensions were pooled and pelleted by centrifugation at 800 g for 5 min at RT and the pellet was re-suspended in complete CPEC medium, which is CPEC medium supplemented with 5 μg/ml human insulin (Sigma Aldrich, St. Louis, MO, USA), 10 ng/ml hEGF (Peprotech, Rocky Hill, NJ, USA), 2 μg/ml hydrocortisone (Sigma, Buchs, Switzerland), 5 ng/ml bFGF (Sigma, Buchs, Switzerland) and 20 μM cytosine arabinoside (Ara-C) (Sigma, St. Louis, MO, USA). To allow for further enrichment of CP epithelial cells by differential attachment to plastic, cells were resuspended in 4 ml of complete CPEC growth medium and plated on 2 non-coated petri dishes (PD35), (Becton–Dickinson Biosciences (BD Biosciences), Franklin Lakes, NJ, USA). After 2–3 h of incubation at 37 °C and 7 % CO_2_, adherent fibroblasts and macrophages, characterized by their extension of cellular processes, were visible under the microscope as described [20]. The supernatant was collected and pelleted for 5 min at 800 g. The single cell pellet was re-suspended in 0.5 ml complete CPEC growth medium and the cells were counted using a hemocytometer. 3 × 10^5^ cells/cm^2^ were plated either on permanox 8-well chamberslides (Thermo Fischer Scientific, Rochester, NY, USA) coated with 20 μg/ml laminin (Roche, Mannheim, Germany) for phase contrast microscopy, on laminin coated 0.33 cm^2^ Transwell filters with 0.4 μm pore size (Corning, Kennebunk, ME, USA) for immunofluorescence stainings, or on laminin coated 5 μm pore size Transwell filters (Corning, Kennebunk, ME, USA) for transmigration assays. The cells were allowed to attach to the chamber slides or filter membranes at 37 °C and 7 % CO_2_ for 48 h. On day 2 the cells were washed with DPBS and the medium was exchanged. The cells were grown to confluence for 1 week at 37 °C and 7 % CO_2_ in complete growth medium, which was changed every other day. Continuous presence of the DNA synthesis inhibitor Ara-C specifically suppressed the growth of contaminating cells such as stromal fibroblasts. In contrast to CPECs, these cells express nucleoside transporters unable to distinguish ribose and arabinose residues and thus incorporate Ara-C into their genomic DNA, leading to the specific suppression of their growth [30, 35].

### Establishment of inducible CP epithelial cell lines from the Immortomouse^®^

Immortomice^®^ were obtained from Charles River Laboratories (Wilmington, MA, USA). Immortomice^®^ are transgenic mice with a thermolabile Simian Virus 40 large T Antigen (SV40 TAg) and limited expression of functional SV40 Tag in vivo, e.g. at 37 °C [36]. At the same time, expression of the thermolabile SV40 TAg under the control of the mouse major histocompatibility complex class I allows for further induction of expression by interferonγ (IFNγ). CPECs were isolated as described above and grown under non-permissive conditions (37 °C and in the absence of IFNγ). Upon reaching confluence, CPECs were passaged and shifted to permissive conditions (33 °C and 10 U/ml IFNγ (PeproTech, Rocky Hill, NJ, USA), allowing for increased expression of functional SV40 TAg. Confluent cell layers were split for up to 6 passages under permissive conditions accompanied by shifting aliquots of the cultures to non-permissive conditions at 37 or 39 °C and IFNγ withdrawal. The latter were tested for epithelial re-differentiation by evaluating their epithelial morphology and by immunostaining for epithelial-specific molecules. In order to establish conditionally immortalized cells, this standard procedure was modified several times at every step, as summarized in Table 1.

**Table 1:** Culture options tested for the establishment of conditional immortoCPEC lines

Source (Immortomice^®^)			
Age	Sex	Genotype	Number of mice per preparation
2–12 weeks, 20 weeks	Female or male	tsSV40Tag heterozygous/homozygous	2 or 8–16

Primary culture (33 or 37 °C)					
Handling options				Culture media supplements	
Seeding density	Laminin coated surface	Duration until passaging and/or temperature switch	Splitting ratio to passage 1	Fetal bovine serum (FBS)	Cytosine arabinoside (AraC)
0.5 × 10^5^–4.5 × 10^5^ cells/cm^2^ 1 × 10^5^ cells/filter limiting dilution cloning (1 cell/0.33 cm^2^)	Petridishes 35 or 100 mm^2^ 8 well chamberslide 48 well plate 96 well plate	0–6 days 29/36 days (limited dilution)	1:2 1:3 1:5 Colony picking	2 % in the absence of fibroblast growth factor (FGF) 10 % (±FGF)	Days 0–4

De-differentiation under permissive conditions (33 °C + IFNγ)						
Handling options		Culture media supplements				
Temperature switch	Splitting ratios	Conditioned medium	IFNγ	Fiborblast growth factor (FGF)	Fetal bovine serum	AraC
With/without splitting of cells	1:2 1:3 1:4 1:5 1:8 limiting dilution cloning	±addition of supernatant from HIBCPPs (human choroid plexus papilloma *cell* line)	0 U/ml 5 U/ml 10 U/ml 30 U/ml 50 U/ml	± FGF	2 % 5 % 10 %	±AraC days 0–4

Re-differentiation under non-permissive conditions (37 or 39 °C)	
Handling options	Culture media supplements
Culture duration	AraC
7, 10, 14 days	day 0–4

### Culture of the ECPC4 cell line

The mouse carcinoma cell line ECPC4 [34] was purchased from the RIKEN BioResource Center (3-1-1 Koyadai, Tsukuba, Ibaraki 305-0074, Japan). ECPC4 cells were received at passage 37 and used for experiments until passage 41. ECPC4 cells were cultured at 37 °C and 7 % CO_2_ in 5 ml growth medium (RPMI 1640 (Gibco, Paisley, UK)/10 % FBS (Gibco, Paisley, UK) in T25 flasks and split in a 1:4 ratio twice per week as described by the distributor.

### Establishment of ‘inverted’ CPEC cultures

ECPC4 and pmCPECs were cultured on the lower (‘inverted’) side of filter inserts using a modification of a protocol previously established for porcine and human in vitro models of the BCSFB [37]. In brief, CPECs were seeded in a volume of 100 μl on the lower side of laminin coated Transwell filters placed in inverted position in 12-well plates. The cells were seeded at the same density as on standard filter sides. After incubation for 24 and 48 h for ECPC4 and pmCPECs, respectively, the cells were washed twice with DPBS and the filters were flipped and placed into their original 24-well plate filled with 0.5 and 1 ml of growth medium per well in the upper and lower compartment, respectively.

### Antibodies and buffers for immunofluorescence staining

The following primary antibodies were used for immunofluorescence staining: monoclonal mouse anti-pan Cytokeratin (mixture) antibody (1:100), (Sigma, C2562, St. Louis, MO, USA), monoclonal mouse anti-human E-cadherin (BD Biosciences, Franklin Lakes, NJ, USA, catalog number 610181, 10 μg/ml), monoclonal mouse anti-β-catenin (BD Biosciences, Franklin Lakes, NJ, USA, catalog number 610154, 10 μg/ml), polyclonal rabbit anti-human transthyretin (TTR) (Dako, Glostrup, Denmark, catalog number A0002, 4.5 μg/ml), polyclonal rabbit anti-claudin-1 (Cldn-1) (Invitrogen, Camarillo, CA, USA, catalog number 519000, 5 μg/ml), polyclonal rabbit anti-claudin-2, or -3 or -11 (Invitrogen, Camarillo, CA, USA, catalog numbers 516100, 341700, 364500, respectively, 10 μg/ml), polyclonal rabbit anti-zonula occludens 1 (ZO1) (Invitrogen, Camarillo, CA, USA, catalog number 617300, 5 μg/ml), and polyclonal goat anti-huVimentin (1:20, Chemicon International Inc. Temecula, CA, USA, catalog number AB1620). Rat anti-mouse ICAM-1 (25ZC7) and rat anti-mouse VCAM-1 (9DB3) were used as hybridoma culture supernatants.

The following 2nd stage antibodies were used: Cy3 donkey anti-mouse IgG (H + L) (Jackson ImmunoResearch, West Grove, PA, USA, catalog number 715-165-151, 2.5 μg/ml), Alexa488 goat anti-rabbit IgG (H + L) (Invitrogen, Eugene, OR, USA, catalog number A11034 10 μg/ml), and Cy3 donkey anti-goat IgG (H + L) (Jackson ImmunoResearch, West Grove, PA, USA, catalog number 705-165-147, 7 μg/ml), and Cy3 donkey anti-rat IgG (H + L) (Jackson ImmunoResearch, West Grove, PA, USA, catalog number 712-165-150, 2.5 μg/ml). The following buffers were made in house: 10 × TBS (50 mM Trizma Base (Sigma, St. Louis, MO, USA), 150 mM NaCl (Sigma, Buchs, Switzerland), 1 mM CaCl_2_·2H_2_O (Sigma St. Louis, MO, USA), adjusted to pH 7.4).

### Immunofluorescence staining

Confluent cell layers grown on Transwell filters (Corning, Kennebunk, ME, USA) were gently washed with warm DPBS and subsequently fixed with either 1 % paraformaldehyde (PFA, MERCK, Darmstadt, Germany) in DPBS at RT for 10 min or with −20 °C methanol (for claudin and occludin staining) for 1 min. After fixation, the cells were washed 5× with DPBS. Blocking solution (5 % skimmed milk (Rapilait, Migros, Switzerland), 0.3 % Triton-X-100 (Schweizerhall, Basel, Switzerland Batch Nr. A11021), 0.04 % NaN_3_ (Fluka Chemie, Buchs, Switzerland) in TBS pH 7.4) was incubated for 20 min at RT. Subsequently 35 μl of blocking buffer containing the 1st antibody was added for 1 h at RT. The primary antibody was removed from the cell layer by 5 washing steps with DPBS and 35 μl of blocking buffer containing the 2nd antibody was added to the filter inserts and incubated light protected for 45 min at RT. The cells were washed 5× with DPBS and incubated under light protection with 1 μg/ml 4′,6-diamidin-2-phenylindol (DAPI, AppliChem, Darmstadt, Germany) in DPBS for 2 min at RT. After washing 3× with DPBS, the cell carrying filters were cut out from the inserts, plated on glass-slides and mounted with embedding medium Mowiol (Sigma-Aldrich, Steinheim, Germany) prior to fluorescence microscopy. Immunostaining for cell surface ICAM-1 and VCAM-1 was performed on live cells. To this end live cells were incubated with the hybridoma culture supernatants for 20 min at RT. After washing with DPBS, the cells were fixed with 1 % formaldehyde in PBS for 5 min at RT, washed with DPBS for 10 min at RT and then incubated with the secondary antibody for 20 min at RT in the dark. After washing 3× with DPBS, the cell-carrying filters were cut out from the inserts, plated on glass-slides and mounted with embedding medium Mowiol prior to fluorescence microscopy.

### Transepithelial electrical resistance

The transepithelial electrical resistance (TEER) across the CPEC monolayers was analyzed in triplicate by impedance spectroscopy employing the cellZscope R (Nanoanalytics, Muenster, Germany) according to the manufacturer’s instructions. To this end cells were seeded on laminin coated 0.4 μm poresized filter inserts (Corning, Kennebunk, ME, USA or Greiner bio-one, Frickenhausen, Germany) in standard or inverted culture as described above. Upon complete pmCPECs attachment after 48 h, the filters were turned upside down and the medium was added to both compartments of the filter system allowing for further proliferation of the cells. The pmCPECs were transferred into the cellZscope and the TEER was measured over the last 72 h in culture to monitor the achievement of the resistance plateau. The ECPC4 cells were split in a 1:4 ratio as recommended by the distributor without counting the cells, and plated on standard and inverted sides of laminin coated Transwell filters. Upon attachment of ECPC4 cells to the porous membranes after 24 h, the filters were transferred into the cellZscope where they grew to confluence for further 3 days. One point TEER measurement of titrated and serum-depleted ECPC4 cells was performed every 24 h using the EndOhm-6 device (World Precision Instruments, Inc. Sarasota, FL, USA). Therefore, the filter inserts were transferred one by one into the chamber containing pre-warmed culture medium. In order to obtain accurate results, hydrostatic pressure on the membranes and air bubble formation were carefully avoided. The electrode was centered within the chamber and the TEER was measured using the Millicell Electrical Resistance System-2 epithelial volt-ohm meter (Millipore Corporation, Billerica, MA, USA). The displayed values were divided by the surface area of the filters, in order to yield the net resistances of the cell monolayers. Blank laminin coated inserts were used as empty filter references.

### Transepithelial permeability

Prior to barrier investigations, the cells were seeded on laminin coated 0.33 cm^2^ Transwell (Corning, Kennebunk, ME, USA) filters, pore diameter 0.4 μm, and the pmCPECs and ECPC4 cells were incubated for 48 and 24 h, respectively. Next, both compartments of the Transwell filter system were filled with fresh medium and the cells were cultured for further 5 days (pmCPECs) or 2–3 days (ECPC4). The paracellular permeability across the epithelial monolayers was measured in triplicate exactly as previously described [38, 39]. In brief, prior to experiments, 3 kDa dextran coupled with Alexa Fluor 680 (Molecular Probes, Eugene, OR, USA) was diluted, light protected, in assay medium (DMEM (Gibco, Paisley, UK), 5 % FCS (Gibco, Paisley, UK), 25 mM Hepes (Gibco, Paisley, UK), 2 % l-glutamine Gibco, Paisley, UK) to a final concentration of 10 μg/ml. 600 μl of the dextran free assay medium was added to the bottom of each well of an empty 24-Transwell plate (Corning, Kennebunk, ME, USA). Permeability assays were always performed on the same CPEC cultures previously analyzed for TEER. Filters were washed once with assay medium, and 100 μl of the dextran solution was added to the upper side of the filter inserts, which were subsequently placed into wells containing 600 μl of assay medium. The plate was incubated at 37 °C and the inserts were replaced into wells containing fresh assay medium, avoiding long exposure to light and room temperature every 10 min. 200 μl samples from all lower compartments were transferred to a 96-well plate. The dextran permeability was measured by scanning the 200 μl samples in the 96-well plate by infrared imaging (Odyssey Quantitative Fluorescence Imaging System, LI-COR, Bad Homburg, Germany). The epithelial permeability coefficient (Pe) was calculated using the clearance principle to obtain a concentration-independent transport parameter as previously described in detail [39]. A standard dilution curve (1 μg/ml–0.1 ng/ml) and empty filters were used to obtain the reference permeability of the empty filter inserts. The permeability coefficient for the in vitro BCSFB models was calculated as follows: the slope of the average tracer volume cleared was plotted versus time in order to obtain the linear regression designated as PSt. The slope of the tracer clearance curve of the coated empty filters was indicated as PSf. The permeability surface area product of the epithelial cell monolayer (PSe) was calculated from PSt and PSf: 1/PSe = 1/PSt − 1/PSf. The PSe was divided by the filter surface area, in order to generate the epithelial Pe in cm/min.

The cell layer Pe for Lucifer Yellow (LY) (Sigma, St. Louis, MO, USA)—MW 457.3 g/mol diluted in assay medium—was determined as previously published in [40] according to the permeability assay for 3 kDa described above, with the assay medium being HBSS (1×) without phenol red (Gibco, Paisley, UK), supplemented with 5 % FCS and 25 mM Hepes, the LY solution of 50 μM and the LY standard dilution curve measured for 20–0.05 μM LY. The fluorescence detection was performed using the Tecan Infinite M1000 device and the Tecan i-control software (Tecan Trading AG, Männerdorf, Switzerland). Calculation of the Pe values by this methodology provides numbers to the 8th decimal. Entering these mathematically-correct numbers into the GraphPad Prism 6 Software for statistical analysis provides values to the 3rd decimal. These mathematically correct Pe values are shown in the “Results”.

### T cell migration across the BCSFB in vitro

In order to assure an unhindered passing of immune cells through the filter pores during the transmigration assay, the CPEC cells were seeded on laminin-coated inverted filters with 5 μm pore size. Inverted pmCPEC cultures were stimulated or not with TNFα (PromoCell GmbH, Heidelberg, Germany) (10 ng/ml) or IFNγ (100 U/ml) 16 h prior to the experiment. Encephalitogenic CD4^+^ Th1 effector/memory proteolipid protein (PLP) peptide aa_139–153_ specific T cells (line SJL/PLP7) were cultured as previously described [41]. On the day of the assay the CD4^+^ T cells were collected in migrations-assay medium (MAM: DMEM (Gibco, Paisley, UK), 2 % l-Glutamine, 25 mM Hepes (Gibco, Paisley, UK), 5 % Calf Serum (CS) (Sigma, St. Louis, MO, USA)). pmCPEC inserts were washed with MAM twice before transferred into a new 24-well Costar plate well containing 600 μl of MAM. 100 μl of MAM containing 1 × 10^5^ T cells was added per insert and T cells were allowed to transmigrate for 8 h at 37 °C. Additionally, a triplicate of input cell suspensions containing 1 × 10^5^/600 μl of MAM were pipetted into 24-well plate wells. The number of transmigrated T cells, the number of the cells in input samples and their viabilities were assessed by the CASY Cell counter and analyzer (Schärfe System, Reutlingen, Germany) according to the manufacturer’s instructions. The percentage of migrated T cells was calculated referring to the inputs as 100 %.

### Statistical analysis

One-way ANOVA was performed followed by the Tukey multiple comparison test to compare three or more groups. For comparison of two groups, a Mann–Whitney test was performed. Results were expressed as mean ± SD and a *P* value <0.05 was considered significant. Statistical analysis was performed using the GraphPad Prism 6 software (GraphPad, San Diego, CA, USA).

## Results

### Isolation and culture of highly purified primary mouse choroid plexus epithelial cells (pmCPECs)

**Fig. 1:**
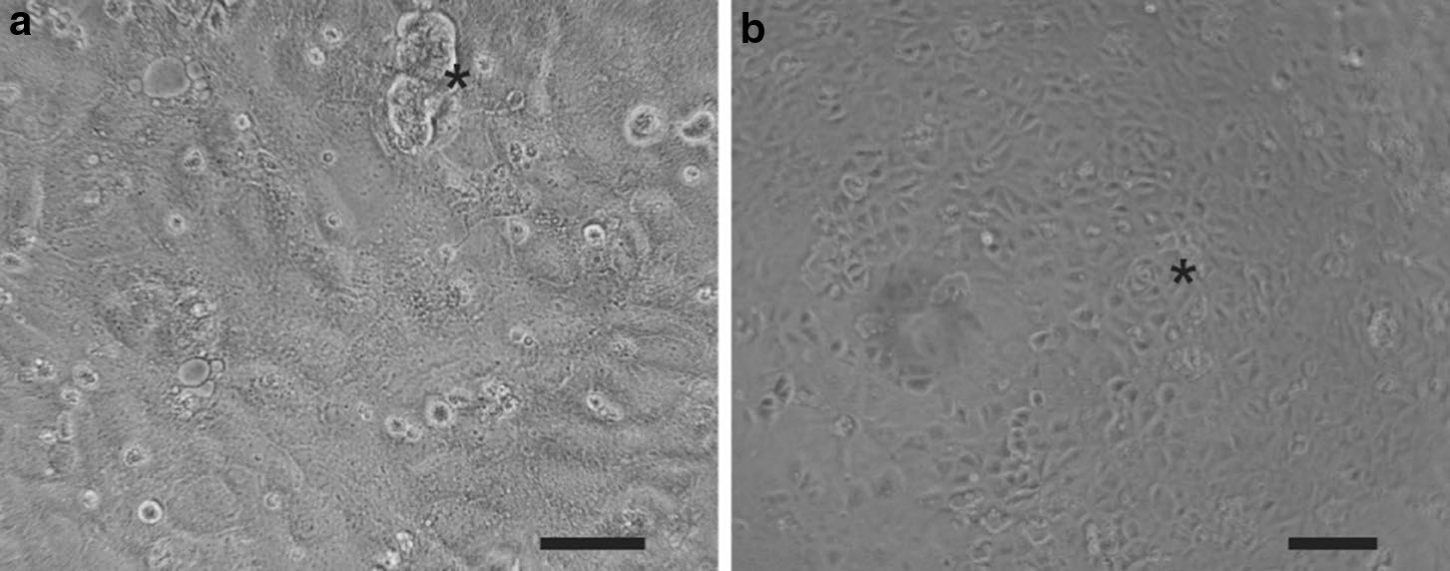
Morphology of confluent primary mouse choroid plexus epithelial cells (pmCPECs). Representative phase contrast pictures of cells plated directly after choroid plexus dissection and cell disaggregation and cultured in complete growth medium for 8 days. The pmCPECs exhibit a predominant polygonal morphology with rare unprocessed tissue remnants (*asterisk*) visible in **a** and occasional dome like cell aggregates (*asterisk*) visible in **b**. The contrast of picture **a** was enhanced using Adobe Photoshop software. *Scale bar* in **a** = 50 μm and in **b** = 100 μm

In order to provide a suitable in vitro model of the mouse BCSFB to investigate the cellular and molecular mechanisms mediating immune cell migration across the BCSFB, we established a procedure for the isolation and culture of highly purified primary mouse choroid plexus epithelial cells (pmCPECs) by adapting a previously-published protocol for the isolation and culture of rat choroid plexus epithelial cells [20]. CPECs were isolated by enzymatic digestion followed by a combined mechanical and enzymatic disaggregation of the choroid plexus from the lateral and 4th ventricles of sex and age matched mice. The preparations yielded 3.3–4.5 × 10^4^ CPECs per mouse. The cells were plated on laminin-coated supports in a density of 3 × 10^5^/cm^2^. The pmCPECs formed islets of cuboidal shaped cells that within 5–7 days grew into confluent monolayers showing contact inhibition (Fig. 1). We did notice the occasional appearance of incompletely processed CP tissue particles (asterisk, Fig. 1a) and the formation of small dome-like epithelial structures after one week of culture (asterisk, Fig. 1b). The high purity of the CPEC culture was confirmed by positive immunofluorescence (IF) staining for cytokeratin in >95 % of cells within the monolayer. Junctional maturation was confirmed by the junctional localization of tight junction proteins, e.g. claudin-1 (e.g. Fig. 4b). Thus our protocol enabled the isolation and growth of highly pure mouse choroid plexus epithelial cells.

### Conditionally immortalized Immortomouse^®^ derived CPEC lines fail to re-differentiate into mature CPECs

Having established primary cultures of pmCPECs, we next aimed to establish conditionally immortalized CPEC lines which would produce proliferating cultures and thus reduce the number of mice needed for the in vitro model. The Immortomouse^®^ that carries the thermo-labile SV40 large T antigen under the control of an IFNγ inducible MHC class I promoter was used [36]. Previous studies have successfully used this mouse model to establish conditionally immortalized epithelial cell lines from other tissues [32].

**Fig. 2:**
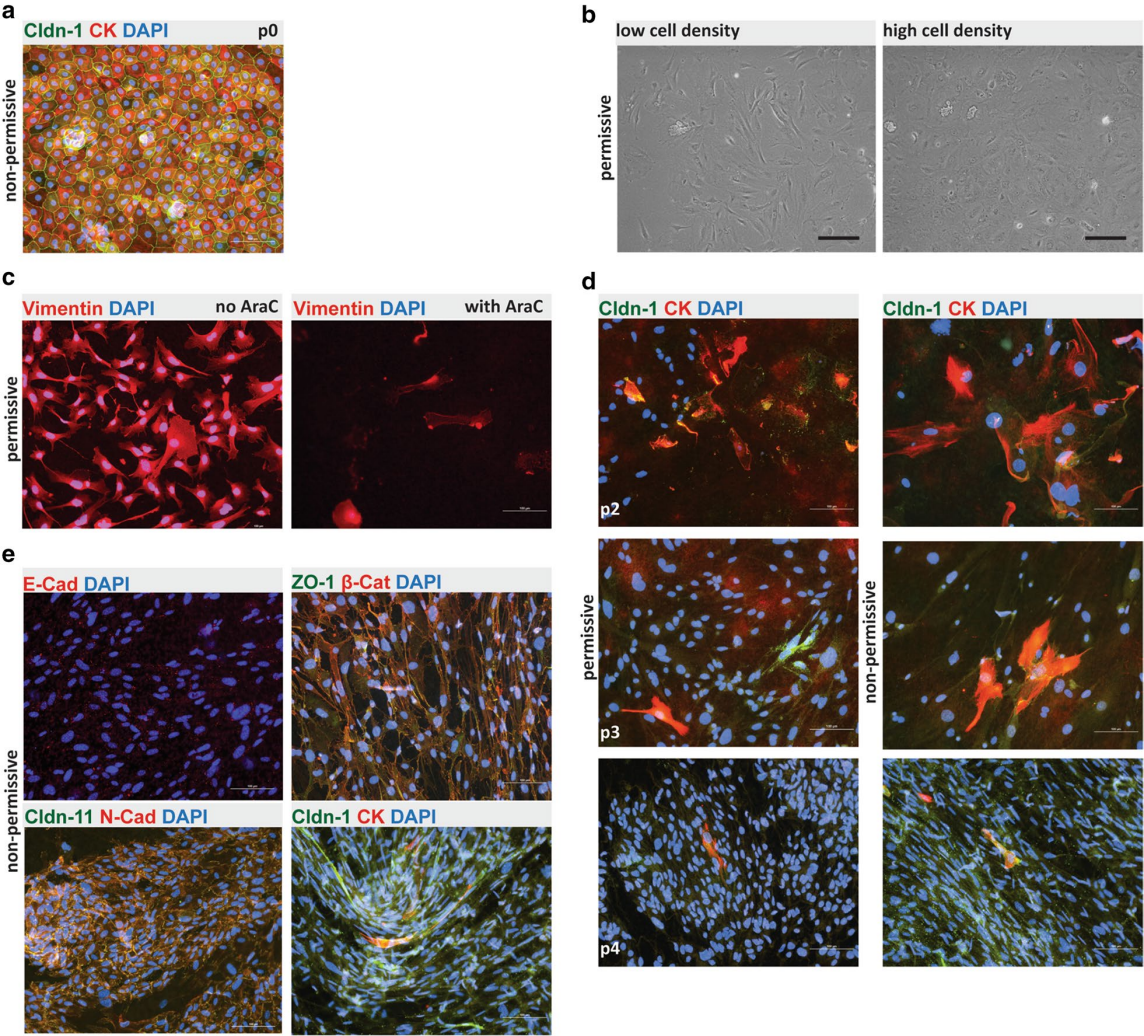
Phenotype and irreversible de-differentiation of Immortomouse^®^ CPECs. **a** Primary Immortomouse^®^ choroid plexus epithelial cells (p0) grown at 37 °C for 7 days showed the same staining patterns for cytokeratin (CK) and claudin-1 (Cldn-1) as pmCPECs isolated from wild-type mice. **b** When Immortomouse^®^ CPECs were grown under permissive conditions (33 °C, 10U IFNγ) the heterogenous de-differentiation process started in areas with low cell density, whereas CPECs kept their cuboidal shape longer in areas with high cell density. The contrast of the pictures in **a** and **b** was enhanced using Adobe Photoshop software. **c** Vimentin staining, rather than CK staining was observed at 33 °C. Cell death took place at 33 °C upon Ara-C addition to culture. **d** The irreversible loss of CPEC specific markers Cldn-1 and CK and an increasing proliferation rate of de-differentiated Immortomouse^®^ CPECs was observed with increased passage. The first row is passage 2 (p2), the second row is p3, the third row is p4. **e** The cells failed to form confluent monolayers or display the correct expression pattern of epithelial markers upon shift to non-permissive temperature and IFNγ withdrawal. Immortomouse^®^ CPECs in **e** were stained after 7 days of non-permissive growth; the passage numbers (p) were E-cad/DAPI: p8, ZO1/β-Cat/DAPI: p5, Cldn11/N-Cad/DAPI: p6, Cldn-1/CK/DAPI: p4. *Scale bar* in all immunofluorescent and phase contrast pictures = 100 μm. *p0* primary culture, *E-Cad* E-cadherin, *N-Cad* N-cadherin, *Cldn-1* Claudin 1, *Cldn-11* Claudin 11, *CK* cytokeratin, *β-Cat* β-Catenin, *ZO1* zona occludens protein 1

When grown under non-permissive conditions (37 °C, no IFNγ) primary cultures of Immortomouse^®^ CPECs were indistinguishable from CPECs of C57BL/6 mice (Fig. 2a). When placed under permissive conditions (33 °C, 10 U IFNγ) the CPECs started to proliferate and rapidly lost their typical cuboidal morphology starting in areas of lower cell density at the edges of the culture dishes while CPECs in the center of epithelial islets kept their cuboidal morphology (Fig. 2b). Immortalized CPECs also lost BCSFB specific expression of receptors and barrier functions as addition of the DNA synthesis inhibitor cytosine arabinoside (Ara-C) induced the cell death of the immortalized CPECs. Furthermore, the cells showed expression of the mesenchymal intermediate filament vimentin (Fig. 2c). Loss of epithelial cell morphology and contact inhibition was accompanied by the continuous loss of expression of cytokeratin as well as loss of expression and junctional localization of E-cadherin and the choroid plexus specific claudins (claudin-1, -2, -3, -11) shown here over several passages (Fig. 2d).

Re-differentiation of the immortalized CPECs was attempted after passages 1–6 at 37 or 39 °C and withdrawal of IFNγ with re-differentiation at 39 °C found to be more effective. Evidence for re-differentiation of the immortalized CPECs into epithelial cells was documented by the re-expression of cytokeratin in a few epithelial cells and the partial reconstitution of AJs (detection of junctional localization of E/N-cadherin and β-catenin) and TJs (detection of junctional localization of ZO-1, claudin-1, claudin-11) (Fig. 2c, e and not shown). Unfortunately this process was limited to small, incoherent islands of epithelial cells and remained incomplete due to the continued expression of vimentin in the cultured cells and cytoplasmic detection of junctional molecules irrespective of passage number (Fig. 2e).

Interestingly, the addition of Ara-C to highly proliferating Immortomouse^®^ CPECs at non-permissive conditions did not induce massive cell death during the re-differentiation time of 7–14 days. However, a selection for re-differentiated cuboidal epithelial cells could not be achieved (data not shown).

In a next step we compared a possible advantage of establishing CPEC lines from Immortomice^®^ homozygous (T/T) or heterozygous (T/+) for the tsSV40Tag. Indeed we found that under permissive conditions Immortomouse^®^ CPECs from mice homozygous for the tsSV40TAg (T/T) proliferated faster than those from heterozygous mice (T/+) (data not shown) but irrespective of their genotype they equally failed to re-differentiate into CP epithelial cells in different passages.

We further tested different concentrations of FBS and found that increasing concentrations of FBS in the CPEC cultures increased the proliferation rate of the immortalized cells, but reducing FBS concentrations under non-permissive conditions did not improve re-differentiation of the CPEC cell lines. Similarly, propagation of the CPEC lines also correlated with the concentration of IFN-γ. When propagating CPEC lines from Immortomice^®^ at 33 °C in the absence or presence of increasing concentrations of IFN-γ we found that in the absence of IFN-γ, CPECs also lost their cuboidal morphology but died after passaging due to their poor proliferation ability. In contrast, increasing concentrations of IFN-γ up to 50 U/ml allowed us to propagate the CPECs over several passages. This supported the notion that the level of SV40Tag expression is critical for cell propagation. However, we did not observe improved re-differentiation of the CPEC lines when propagated in the absence or in the presence of higher concentrations of IFN-γ.

Thus, although we successfully established primary cultures of Immortomouse^®^ CPECs and could readily propagate these cells, a total of 15 different trials applying different handling options and culture media (summarized in Table 1) failed to achieve successful re-differentiation of Immortomouse^®^ CPECs. We therefore concluded that in contrast to other tissues the Immortomouse^®^ is not a good source for establishing in vitro models of the mouse BCSFB.

### Phenotypic characterization of the mouse choroid plexus carcinoma cell line ECPC4

**Fig. 3:**
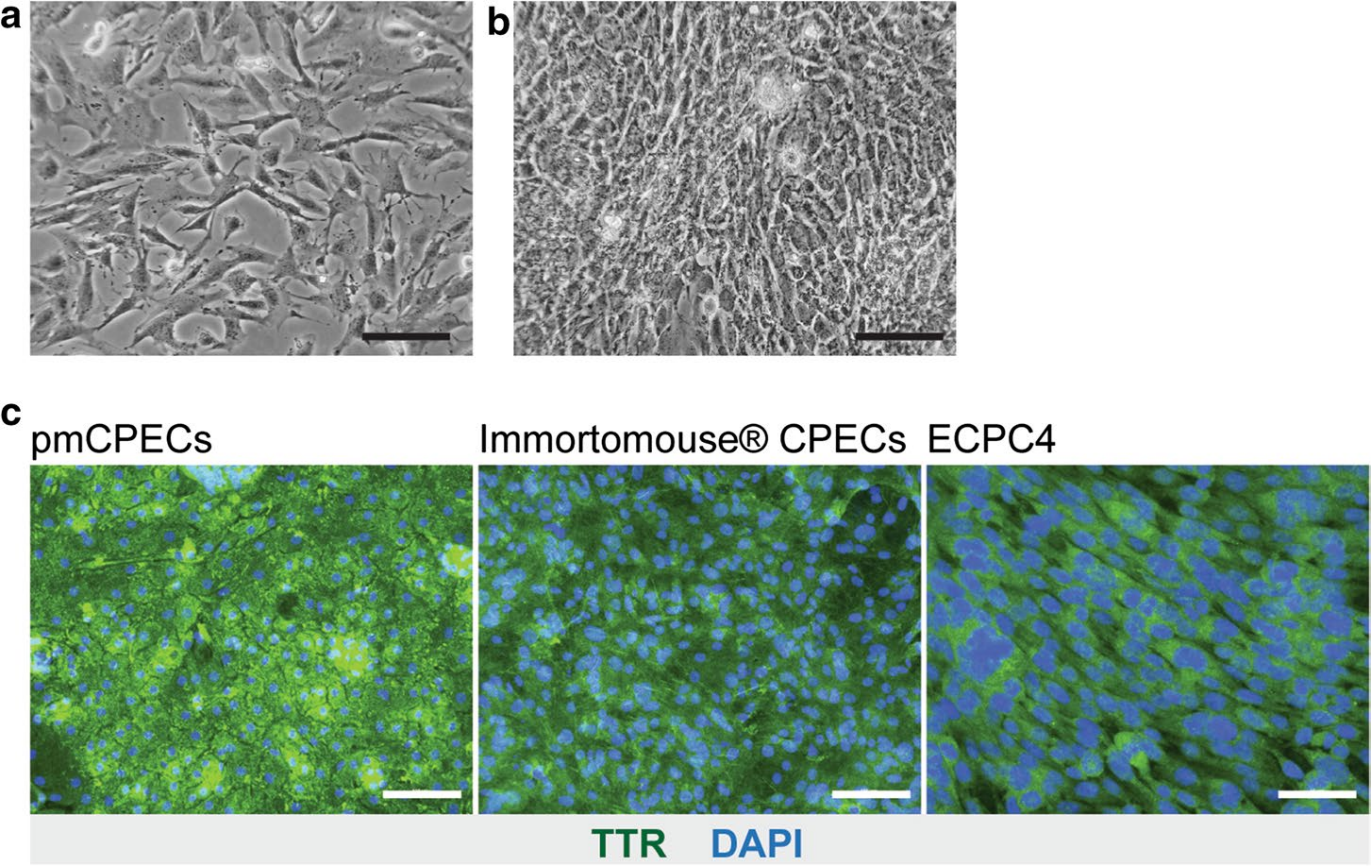
Morphology, expression of transthyretin and propagation of ECPC4 cells. Representative phase contrast pictures of ECPC4 cells from passage 41 on d1 (**a**) and d4 (**b**) after sub-cultured in a 1:4 ratio as described by the distributer. The cells did not show typical epithelial morphology (**a**, **b**) and grew in overlapping layers (**b**). *Scale bars* 50 μm. **c** Immunofluorescence staining for the CPEC-specific marker transthyretin (TTR) is shown in pmCPECs, Immortomouse^®^ CPECs from passage 6, grown for 7 days at non-permissive conditions and for the ECPC4 cells. *Scale bars* **a**, **b** and ECPC4 cells = 50 μm; pmCPECs and Immortomouse^®^ CPECs = 100 μm

Next we investigated the suitability of the commercially available mouse choroid plexus carcinoma cell line ECPC4 as an in vitro model to study immune function of the mouse BCSFB. ECPC4 cells were reported to be a useful model to study the BCSFB in vitro as they retain the characteristics of CPECs as shown by expression of BCSFB-specific proteins such as transthyretin and alpha2-macroglobulin [34]. When cultured according to the protocol of the manufacturer, ECPC4 cells grew with a high proliferation rate starting with a fibroblast like morphology (Fig. 3a) into confluent cell layers showing incomplete contact inhibition and thus partial overgrowth (Fig. 3b). In order to determine if exogenous addition of extracellular matrix proteins would improve monolayer formation by ECPC4, culture dishes were coated with laminin, rat-tail collagen, gelatin or poly-l-lysine on different glass and plastic surfaces. Irrespective of the condition chosen, we failed to observe any change in the proliferation rate or quality of monolayer formation of ECPC4 cells (data not shown).

To establish an in vitro model accessible from both sides and therefore suitable to study the migration of immune cells across the BSCFB, we cultured ECPC4 cells on porous membranes and tested their growth and viability. For our experiments we plated the ECPC4 cells directly on laminin coated filter inserts with a growth area of 0.33 cm^2^ and let them grow to confluence.

**Fig. 4:**
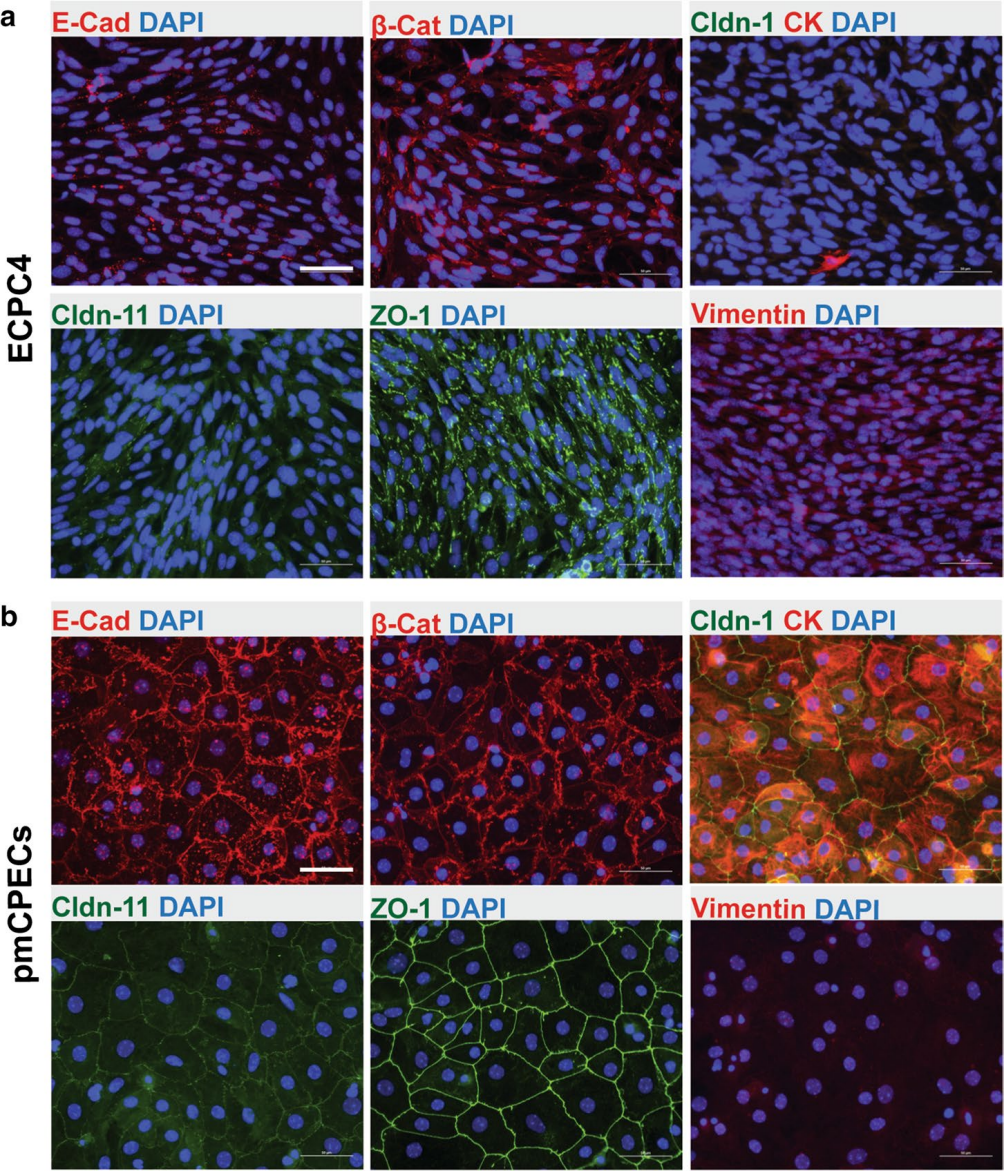
Phenotype of ECPC4 cells and primary mouse choroid plexus epithelial cells (pmCPECs). Immunofluorescence staining for CPEC specific proteins is shown in ECPC4 cells (**a**) and pmCPECs (**b**). **a** There is weak staining for the adhesion junction (AJ) protein E-Cadherin (E-Cad) and its cytoskeleton linker β-catenin (β-Cat) of ECPC4 cells and their localization is not specifically at the plasma membrane. Staining for tight junctional (TJ) claudins-1 and -11 was absent or showed a weak cytosolic pattern, respectively. The scaffolding protein ZO1 staining was disrupted. Additionally, the cell line failed to stain for the early epithelial marker cytokeratin but rather was positive for the mesenchymal intermediate filament protein vimentin. ECPC4 cells from passage 41 were stained on d4 in culture. **b** In contrast, the staining of pmCPECs stained on d7 in culture, revealed a proper distribution of all epithelial markers. pmCPECs. All staining was performed at least 3 times. *Scale bars* 50 μm. *E-Cad* E-cadherin, *Cldn-1* Claudin 1, *Cldn-11* claudin 11, *CK* cytokeratin, *β-Cat* β-catenin, *ZO-1* zona occludens protein 1

To determine their CPEC specific characteristics we next performed immunofluorescence (IF) staining for CPEC specific molecules. We first confirmed expression of transthyretin (TTR), which is known to be highly expressed in the CP in vivo, in the cultured ECPC4 cells in a similar fashion as in Immortomouse^®^ CPECs and pmCPECs (Fig. 3c). However, we could not detect the epithelial cell specific intermediate filament cytokeratin. Rather ECPC4 cells stained positive for the mesenchymal intermediate filament protein vimentin. Staining for the AJ molecules E-cadherin and β-catenin and the TJ proteins occludin, claudin-1, -2, -3 and -11 and ZO-1 showed interrupted junctional localization or no detectable expression, underscoring the immaturity of the junctional complexes between ECPC4 cells (Fig. 4a and data not shown). Thus although ECPC4 cells express CP characteristic proteins they fail to establish contact-inhibited monolayers with mature junctional complexes.

### Phenotypic characterization of primary mouse choroid plexus epithelial cells (pmCPECs)

We next asked if pmCPECs grown on porous filter inserts will form monolayers with intact junctional complexes. We plated the freshly isolated choroid plexus single cell suspensions directly on laminin-coated filter inserts with a growth area of 0.33 cm^2^ to avoid passaging and let the cells proliferate until they attained confluence. Immunofluorescence staining showed expression of TTR (data not shown) and the epithelial cell specific intermediate filament protein cytokeratin in >95 % of cells in the monolayer. In addition we observed continuous junctional staining for the AJ proteins E-cadherin and β-catenin and the TJ proteins claudin-1, -2, -3, -11, occudin and ZO-1 between the pmCPECs (Fig. 4b). Hence the immunostaining confirmed the high purity and the establishment of mature junctional complexes in pmCPEC cultures.

### Comparison of barrier characteristics of ECPC4 and pmCPECs

To determine if the immunofluorescence staining of ECPC4 and pmCPECs correlated with their barrier characteristics we next established for both CPECs the ‘inverted’ culture as previously described for porcine CPECs [37]. To allow investigation of immune cell migration from the blood to the CSF side, CPECs were seeded on the lower surface of inverted filter inserts, which once the CPECs had attached, were turned around for performing the respective assays. Neither ECPC4 nor pmCPECs showed a difference in growth rate or differentiation when grown on the lower filter site compared to the upper filter site as confirmed by light microscopy and immunofluorescence staining for epithelial and junctional molecules exactly as described above (data not shown).

**Fig. 5:**
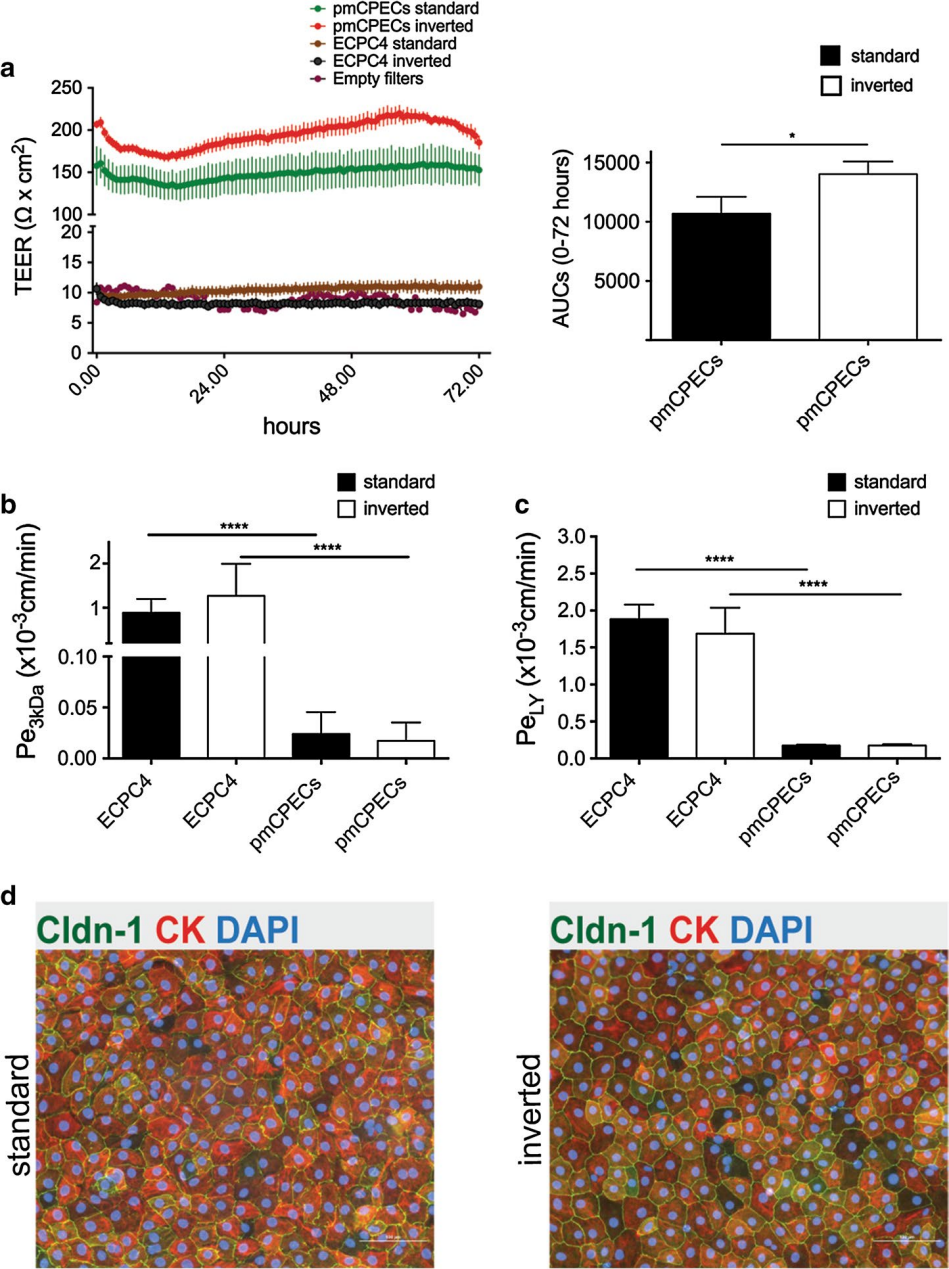
Comparison of barrier characteristics of ECPC4 versus pmCPECs. **a** The time-dependent progression of the transepithelial electrical resistance (TEER) of ECPC4 cells and pmCPECs grown on standard (luminal) or inverted (abluminal) Transwell filter inserts was measured by impedance spectroscopy using the cellZscope device. The TEER of ECPE4 hardly differs from the TEER measured across laminin coated empty filters (EF). In contrast, pmCPECs reach a TEER of 150–200 Ω cm^2^ on d7. The figure shows one representative experiment (of 4) of pmCPECs in comparison to ECPC4 over their last 72 h in culture with 3 filters per condition and 1 empty filter. The *colored lines* show the mean TEER values of triplicate measurements surrounded by *colored* areas, which represent the SD. The area under the *curve* (AUC) as a measure for the overall TEER across the cellular barriers over time (Unit: Ω cm^2^ h) was assessed for a comparison of the overall resistance of the cell layers. *p < 0.05. **b**, **c** The permeability for Alexa Fluor 680-3 kDa dextran (Pe_3kDa_) (**b**) was measured in 5 independent experiments with at least three filters per condition (ECPC4 standard: n = 3, ECPC4 inverted: n = 3, pmCPECs standard: n = 5, pmCPECs inverted n = 4) and the permeability for 457 Da Lucifer Yellow (Pe_LY_) was measured once with at least 3 filters per condition (ECPC4 standard: n = 3, ECPC inverted: n = 3, pmCPECs standard: n = 5, pmCPECs inverted: n = 4) ****p < 0.0001 (**c**). **d** Immunofluorescence staining for claudin-1 (Cldn-1), cytokeratin (CK) and nuclei (DAPI) showed no differences between monolayers grown on the upper (standard) or lower (inverted) side of the filter. *Scale bar* 100 μm. *Bars* in **b**, **c** represent the mean permeability coefficients Pe ± SD

To determine the efficacy of the pmCPECs and the ECPC4 cell line to form a tight barrier with sealing junctional complexes between adjacent cells, we measured the TEER of the cultured cells over the last 72 h in culture to monitor the achievement of the resistance plateau by impedance spectroscopy. In parallel, the TEER was measured over empty laminin-coated filters as negative controls. After 7 days in culture, pmCPEC layers reached a TEER plateau between 100 and 200 Ω cm^2^ (Fig. 5a), which is similar to TEER values previously measured across rat CPE monolayers, being 178 Ω cm^2^ [20] and are slightly higher than those previously observed across mouse CPECs [8]. Interestingly, the inverted pmCPEC cultures displayed a significantly higher TEER than the standard cultures, shown by the calculated area under the curve (AUC) (Units: Ω cm^2^ h) (Fig. 5a). However, the morphology of the pmCPECs growing on either side of the filter was indistinguishable (Fig. 5d).

Irrespective if grown in standard or inverted filters, the ECPC4 cells failed to establish a barrier with regard to TEER measurements. With 10 Ω cm^2^ the TEER measured across ECPC4 monolayers remained close to the TEER values measured across laminin-coated empty filters (Fig. 5a).

The barrier characteristics of the CPEC monolayers were investigated by measuring the paracellular diffusion of the small water soluble tracers 3 kDa dextran and Lucifer Yellow (LY, MW 457 Da) across the epithelial monolayers for 40 min. Monolayers layers of pmCPEC established a tight barrier with permeability coefficients Pe_3kDa_ = 0.024 ± 0.021 × 10^−3^ cm/min and Pe_3kDa_ = 0.017 ± 0.018 × 10^−3^ cm/min for standard and inverted filter cultures, respectively (Fig. 5b). As expected, the permeability for the small tracer LY was higher than that observed for the larger 3 kDa dextran with Pe_LY_ = 0.173 ± 0.014 × 10^−3^ cm/min and Pe_LY_ = 0.173 ± 0.021 × 10^−3^ cm/min for standard and inverted filter pmCPEC cultures, respectively (Fig. 5c).

To compare pmCPEC barrier characteristics with those of ECPC4 monolayers, diffusion of 3 kDa dextran and LY was assessed in the same assays for the ECPC4 in parallel and found to be significantly higher than the Pe values measured for pmCPECs. Specifically, the permeability coefficient for 3 kDa dextran across ECPC4 was Pe_3kDa_ = 0.889 ± 0.311 × 10^−3^ cm/min and Pe_3kDa_ = 1.272 ± 0.720 × 10^−3^ cm/min for standard and inverted filter cultures, respectively (Fig. 5b). For LY, the permeability coefficients were Pe_LY_ = 1.883 ± 0.198 × 10^−3^ cm/min and Pe_LY_ = 1.687 ± 0.351 × 10^−3^ cm/min for standard and inverted filter cultures respectively (Fig. 5c). Thus, in contrast to pmCPECs ECPC4 failed to establish a tight barrier model in vitro.

**Fig. 6:**
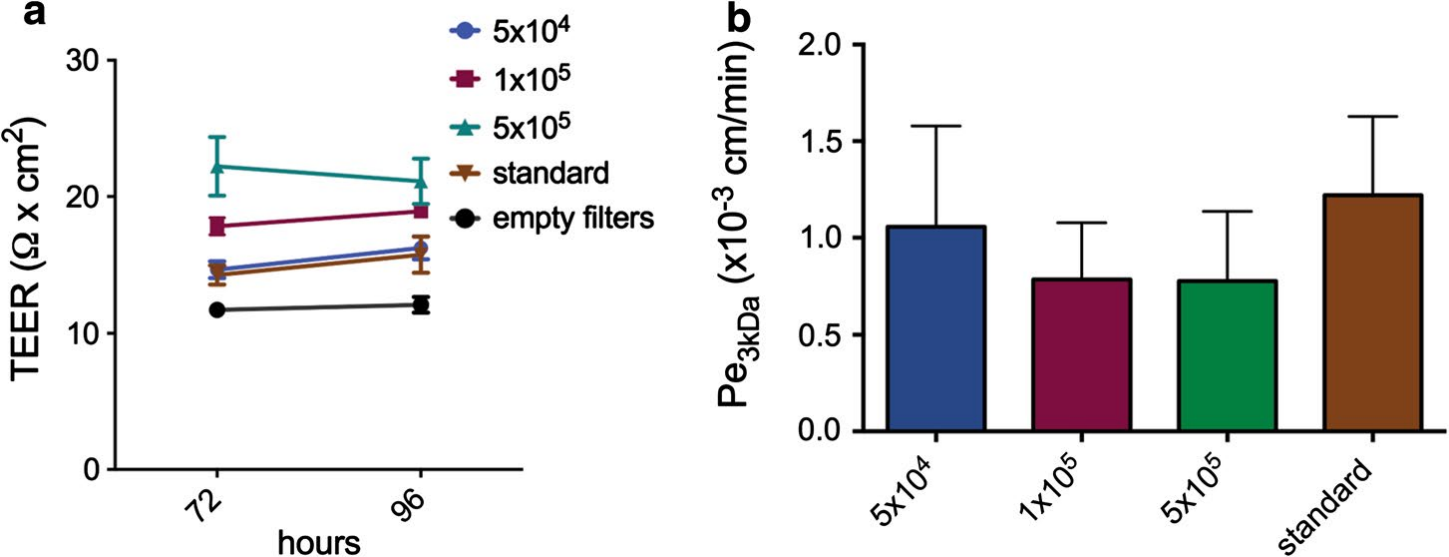
Barrier properties of titrated ECPC4 cells growing on Transwell filter inserts. **a** Different numbers of ECPC4 cells (5 × 10^4^, 1 × 10^5^, 5 × 10^5^ and standard) were plated on the inverted porous filter membranes in triplicate. Standard = ECPC4 continuously split in a ratio 1:4, according to the distributer’s protocol (number of cells not counted). TEER was measured manually once per day on d3 and d4 in culture using the EndOhm device. The cells failed to build up a resistance irrespective of their seeding density. **b** The permeability coefficient (Pe) for Alexa Fluor 680–3 kDa dextran was assessed after the resistance measurement on d4. *Bars* represent the mean ± SD of all filters from two independent experiments, n = 6 for all groups

Considering the different growth characteristics of ECPC4 versus pmCPECs we asked if optimizing the growth conditions of the ECPC4 cells on the filter inserts might improve their barrier characteristics. To this end we performed a titration of the ECPC4 cells on the laminin-coated filters to optimize their seeding concentration. ECPC4 cells were plated in two experiments at different concentrations (5 × 10^4^, 1 × 10^5^ and 5 × 10^5^ cells per filter) and compared to the standard recommended by the distributor (ECPC4 split in a 1:4 ratio, concentration not known) and once at a lower concentration (1 × 10^4^ cells per filter). The TEER of the ECPC4 cell layer was measured manually using the EndOhm device once per day on d3 and d4. The TEER of the cell layers with the maximum value being 22 Ω cm^2^ was slightly higher than that of the empty filters with 12 Ω cm^2^ (Fig. 6a). Next, we investigated the permeability of these titrated cells for 3 kDa dextran on day 4 in culture. Despite different seeding concentrations, the permeability coefficient for the ECPC4 cell layers was however similar, ranging from Pe_3kDa_ = 1.220 × 10^−3^ cm/min to Pe_3kDa_ = 0.775 × 10^−3^ cm/min (Fig. 6b). The ECPC4 plated at the lower concentration of 10^4^ cells per filter showed an even higher Pe_kDa_ = 3.340 × 10^−3^ cm/min (data not shown). Immunofluorescence staining of the filters for ZO-1 and F-actin binding rhodamine phalloidin following the permeability measurements, confirmed closed monolayers without contact inhibition with higher and lower cell numbers (data not shown). Thus, changing the number of ECPE4 growing on filter inserts did not improve the barrier characteristics of these cells.

To change cell-substrate contacts as previously reported [42] we next investigated if ECPC4 grown on polyester (PET) instead of polycarbonate filter inserts have improved barrier properties and failed to find any improvement (data not shown). Finally, as serum withdrawal from the culture medium has been reported to result in improved barriers of porcine CPECs [15] we investigated if ECPC4 barrier properties improve after serum withdrawal from d2 to d4 after seeding. Serum withdrawal did not affect the monolayer integrity, however, it also failed to improve barrier characteristics of ECPC4 when compared to those continuously grown in 10 % FBS (data not shown). In summary, we conclude that in contrary to the commercially available ECPC4 cell line, the pmCPEC cultures form a tight cellular barrier when grown on filter inserts and are thus suited as in vitro model for the mouse BCSFB.

### Comparison of barrier characteristics of pmCPECs on 0.4 and 5 μm pore sized filters

Having established a tight in vitro model of the mouse BCSFB we next adapted this model to the growth on inverted filters with a larger pore size of 5 μm allowing immune cells to reach the pmCPEC monolayer from the upper filter compartment.

**Fig. 7:**
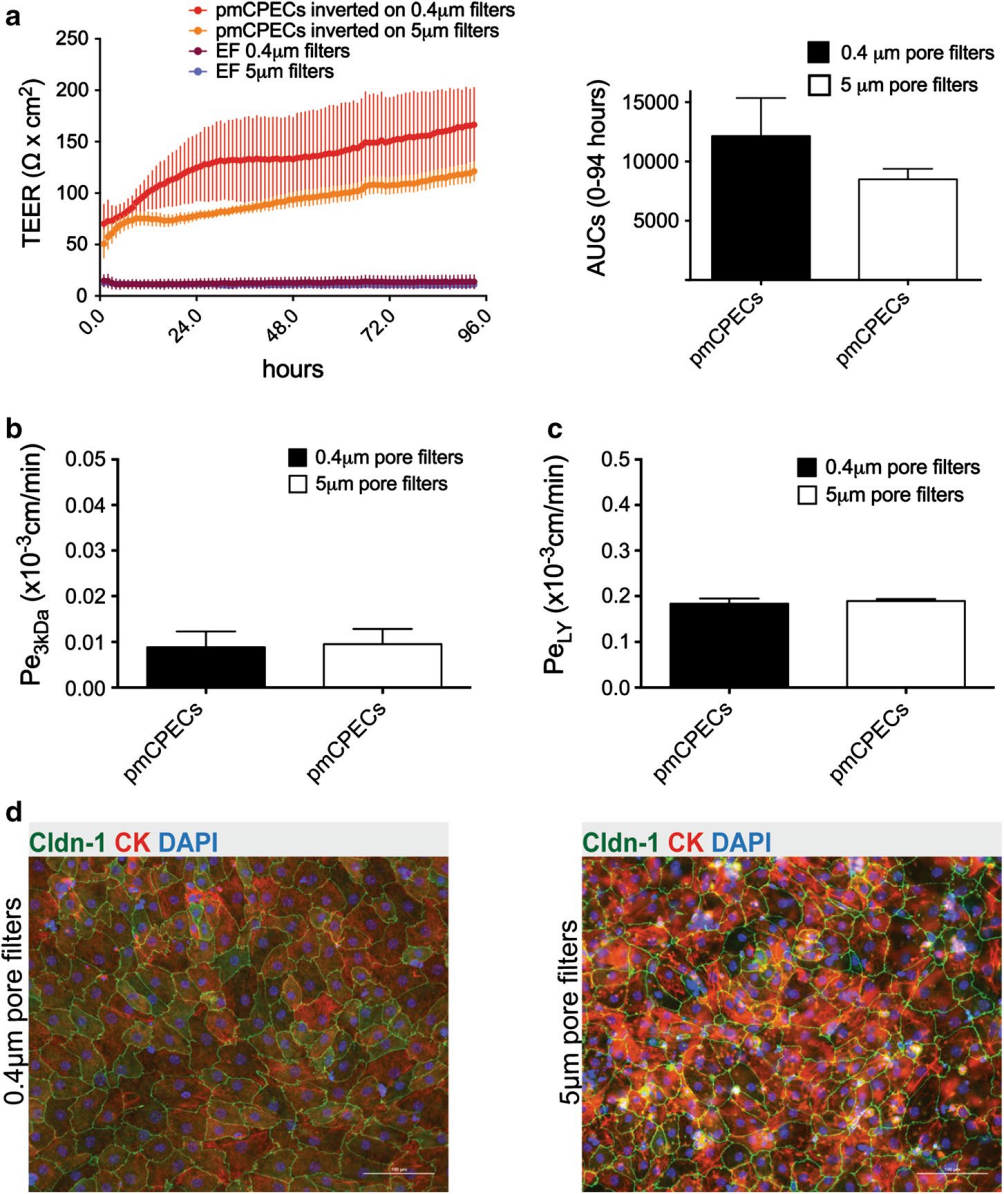
Comparison of barrier characteristics of inverted cultures of pmCPECs on filters with 0.4 and 5 μm pores. **a** The time dependent progression of the transepithelial electrical resistance (TEER) of pmCPECs grown or inverted (abluminal) Transwell filter inserts with 0.4 and 5 μm pores was measured by impedance spectroscopy using the cellZscope device from d3 to d7 in culture. The pmCPECs on both kinds of filters reached a TEER of 100–150 Ω cm^2^ on d7. The figure shows one experiment with 3 filters per condition and 3 empty filters per condition. The *colored lines* show the mean TEER values of triplicate measurements surrounded by *colored* areas, which represent the SD. The area under the curve (AUC; Units: Ω cm^2^ h) was assessed for a comparison of the overall resistance of the cell layers over time with no significant difference detected. **b**, **c** The permeability of the pmCPEC grown on inverted Transwell filter inserts with 0.4 and 5 μm pores for two different small molecular tracers was determined following the TEER measurements in 1 experiment with n = 4 filters per condition. There was no difference for the permeability of Alexa Fluor 680–3 kDa dextran (Pe_3kDa_) (**b**) or for 457 Da Lucifer Yellow (Pe_LY_) (**c**) across the pmCPECs cultured on either type of filter. **d** Immunofluorescence staining for claudin-1 (Cldn-1), cytokeratin (CK) and nuclei (DAPI) on pmCPEC monolayers grown on the inverted sides of filter inserts with 0.4 and 5 μm pores. The difference in clarity between the pictures is due to different microscopic characteristics of the filters. *Scale bar* 100 μm. *Bars* represent the mean permeability coefficients Pe ± SD

In order compare barrier integrities of pmCPECs grown to confluence on 5 μm pore versus 0.4 μm pore inverted filters, the time-dependent progression of the TEER was measured by impedance spectroscopy using cellZscope from d3 to d7 of culture (Fig. 7a). Monolayers of pmCPEC reached TEER values between 100 and 150 Ω cm^2^ on day 7 of culture. Although the TEER of pmCPECs grown on 0.4 μm pore filters seemed apparently higher, this difference was not significant, as shown by the calculated AUC of the TEER over time. Furthermore, the permeability of the pmCPEC grown on filter inserts with 0.4 and 5 μm pores for the Alexa Fluor 680–3 kDa dextran (Pe_3kDa_) and 457 Da Lucifer Yellow (Pe_LY_) (Fig. 7b, c) was comparable. The permeability coefficient for 3 kDa dextran was Pe_3kDa_ = 0.009 ± 0.003 × 10^−3^ cm/min and 0.010 ± 0.003 × 10^−3^ cm/min for the pmCPECs grown on filters with 0.4 and 5 μm pores, respectively. The permeability coefficient for Lucifer Yellow (Pe_LY_) was Pe_LY_ = 0.183 ± 0.011 × 10^−3^ cm/min and Pe = 0.189 ± 0.004 × 10^−3^ cm/min for the pmCPECs grown on filters with 0.4 and 5 μm pores, respectively. Finally, we confirmed comparable growth of contact inhibited monolayers of highly pure pmCPECs on the inverted sides of filter inserts with 5 and 0.4 μm pores by performing immunostainings for claudin-1 (Cldn-1) and cytokeratin (CK) (Fig. 7d). In summary, inverted filter cultures of pmCPEC could successfully be adapted to growth on 5 μm pore Transwell filters without impairing their barrier characteristics.

### Encephalitogenic CD4^+^ T cells migrate across the BCSFB in vitro

**Fig. 8:**
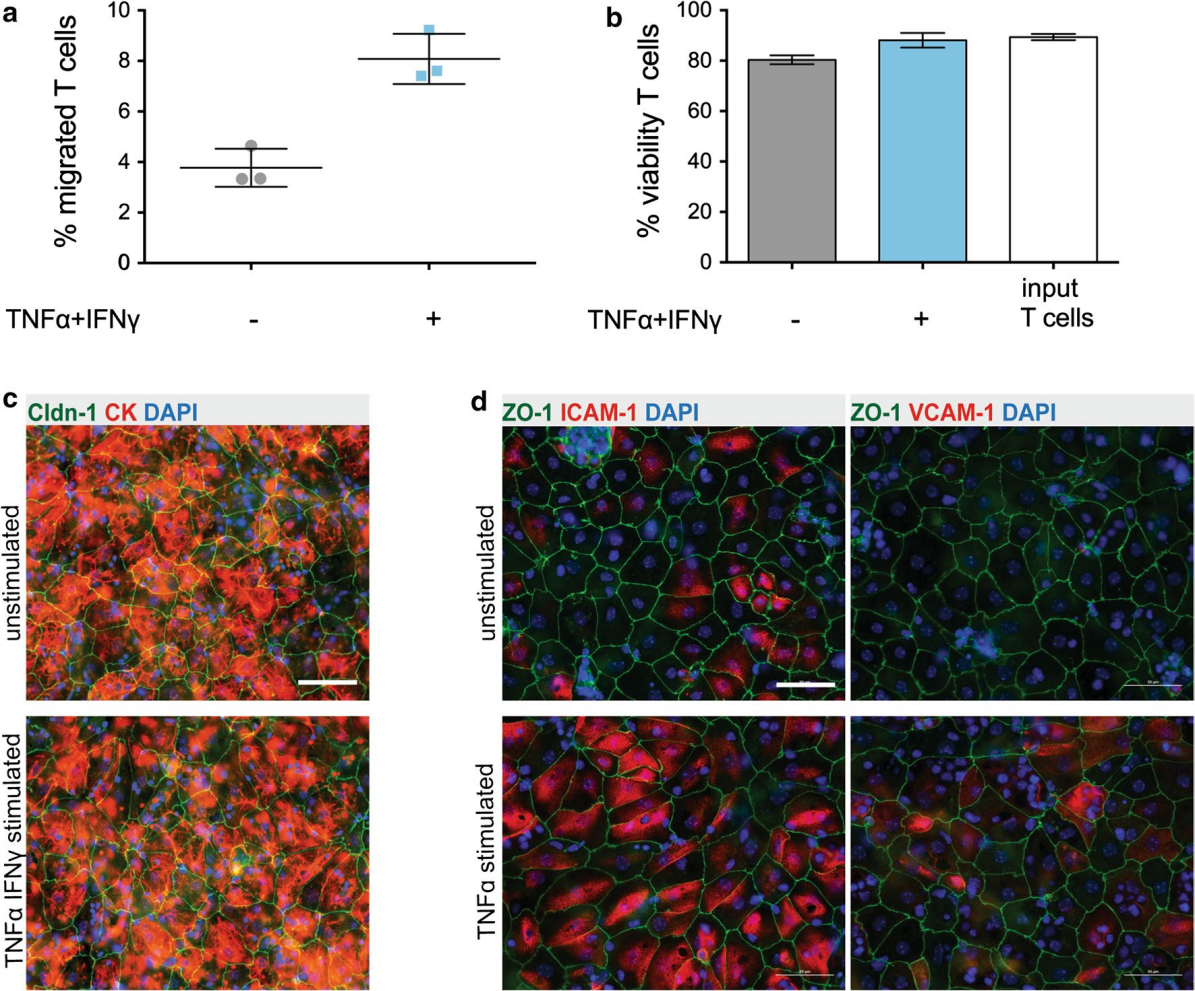
Migration of encephalitogenic CD4^+^ Th1 cells across the BCSFB in vitro. **a** Transmigration rates of encephalitogenic CD4^+^ Th1 effector/memory T cells across non-stimulated and cytokine-stimulated CPECs during a period of 8 h were assessed in vitro. Percentage of total transmigrated encephalitogenic T cells across the unstimulated (−) and TNFα/IFNγ co-stimulated (+) pmCPEC layer in relation to the input sample referred as 100 %. Data represent mean ± SD of one experiment with three filters per condition. **b** Viability of T cells in the lower compartment of the Transwell filter system was confirmed after the incubation time of 8 h. The *error bars* represent mean ± SD. **c** Immunofluorescence staining for the TJ protein Claudin 1 (Cldn-1) and for cytokeratin (CK) confirming the intact cellular monolayer integrity after the transmigration assay. **d** Immunostaining of pmCPECs showing upregulation of adhesion molecules ICAM-1 and VCAM-1 upon stimulation with the pro-inflammatory cytokine TNFα. *Scale bars* in **c** and **d** = 50 μm

Having ensured that barrier characteristics of the mouse BCSFB were maintained on 5 μm pore Transwell filters we next asked if encephalitogenic T cells could migrate from the blood to the CSF side across this pmCPEC monolayer. For this, the pmCPECs were stimulated or not with a combination of TNFα and IFNγ. Indeed we found that a small percentage of encephalitogenic T cells (3.8 %) could cross the non-inflamed pmCPEC monolayer by reaching the lower part of the chamber system after 8 h of incubation time. Stimulating the pmCPECs with a combination of TNFα and IFNγ to inflammation doubled T cell migration across the pmCPEC monolayers (8.1 %) within the same time (Fig. 8a). In contrast, spontaneous migration of encephalitogenic T cells across empty filters during that time amounted to almost 70 %. The viability of the transmigrated T cells was not impaired after crossing the pmCPEC monolayers (Fig. 8b). Similarly, the integrity of the pmCPECs monolayers was not affected by T cell diapedesis as confirmed by immunofluorescence staining for junctional molecules like ZO-1 and claudin-1 and cytokeratin after the 8 h co-incubation of T cells with the pmCPEC monolayers (Fig. 8c and data not shown). Stimulation of pmCPECs with a combination of TNFα/IFNγ induced increased expression of ICAM-1 and VCAM-1 at the protein level (Fig. 8d and not shown) suggesting a potential role of these molecules in the increased migration of T cells across the TNFα/IFNγ stimulated pmCPECs compared to non-stimulated pmCPECs. Thus, encephalitogenic T cells can migrate across our in vitro model of the BCSFB in the absence and presence of inflammatory stimuli. At the same time, this in vitro model of the mouse BCSFB provides a barrier for T cell migration and is thus suitable to study the mechanisms present at the BCSFB that limit immune cell entry into the CNS. Taken together, our in vitro model for the mouse BCSFB constituted of primary mouse CPECs in an inverted filter culture is highly suited to study the cellular and molecular mechanisms of T cell migration across this barrier.

## Discussion

There is increasing evidence for an important role of the CP in CNS immunity. Comparative transcriptome analyses of choroid plexus tissue from healthy mice and mice suffering from neuroinflammatory conditions such as EAE showed the strongest upregulation of expression of genes encoding for adhesion molecules, chemokines and cytokines as well as T cell activation markers, supporting a function of the CP in mediating T cell migration into the CNS [43, 44]. To reach the CSF space at this site circulating T cells first have to cross the wall of the fenestrated CP microvessels allowing them to reach the CP stroma. In a next step they have to migrate across the BCSFB established by CP epithelial cells.

To delineate the cellular and molecular mechanisms mediating the migration of immune cells across the BCSFB from those involved at the BBB, suitable in vitro models established from CP epithelial cells are mandatory. Especially porcine and rat in vitro models of the BCSFB have been successfully established however only a few of these studies have specifically addressed the migration of leukocytes across the BCSFB (summarized in [45]).

In light of the fact that transgenic approaches enabling the specific manipulation of individual genes have advanced in mice and have become an irreplaceable tool to analyze gene function in development and pathology, mouse models of the BCSFB could significantly improve investigations on the potential immune functions of the CP. Although a number of laboratories has established in vitro models of the mouse BCSFB [28, 30, 31] these models have not been optimized for the in vitro study of the immune functions including immune cell migration from the periphery into the CNS [8].

Considering the small tissue size of the mouse CP, primary cultures of CPECs come with the obvious disadvantage of the necessity to sacrifice numbers of mice. In addition, primary cultures of CPECs have proven to have a limited life span of 1–2 weeks and have been reported upon passaging to show signs of dedifferentiation, death or fibroblast enrichment [14, 22, 29]. However, the low turnover rate of primary mouse CPECs can be overcome by the addition of growth factors like insulin-like growth factor 1 (IGF-1) and epidermal growth factor (EGF) to the culture [31] implying that optimal culture conditions of these cells allow for appropriate expansion.

Nevertheless appropriate cell line models for the mouse BCSFB would be preferable. Cell lines in general bear the advantage of easy handling. They overcome the disadvantages of primary cell cultures such as repeated necessity to sacrifice significant numbers of mice and their short viability as well as their lack of suitability for high throughput experiments. Establishing cell lines from primary brain endothelial cells has been successfully achieved in the past by expression of the polyoma middle T oncogene [46] or a temperature-sensitive SV40 large T followed by expression of the catalytic subunit of telomerase [47]. In addition, CP tumors have been used to establish CPEC lines. Furthermore, the SV40 large T induced rat CPEC line Z310 establishes with a TEER of 150–200 Ω cm^2^ barrier characteristics similar to those of primary rat CPECs [22]. At the same time, Z310 cells fail to express CPEC specific transporters and junctional proteins [48, 49] limiting its suitability for a number of research approaches. The slowly proliferating human CP papilloma cell line HIBCPP has proven a suitable in vitro model for the human BCSFB. If grown under optimized conditions, HIBCPP build a permeability barrier reaching a TEER of 200–500 Ω cm^2^ and although they only show a partial contact inhibition, HIBCPP cells express the CPEC-specific junctional proteins including claudin-11 as observed in vivo [25, 50].

In the present study we have undertaken elaborate efforts to establish cell line and primary cell models for the mouse BCSFB from different mice that will allow study of the immune functions of this barrier including the molecular mechanisms mediating the migration of immune cells across the BCSFB. Having established primary cultures of mouse CPEC we chose to use the Immortomouse^®^ as a cellular source for the establishment of CPEC lines. Immortomice^®^ express a temperature- sensitive mutant of the SV40 large T antigen tsA58, which supports cell survival and growth by overcoming p53- and pRB-dependent cell cycle arrest, under the control of the MHC class I promoter. These mice have been successfully used to establish epithelial cell lines from the gastrointestinal tissues (summarized in [32]), the kidney [51] and the mammary gland [33] by expanding epithelial cultures under permissive culture conditions, namely at 33 °C and in the presence of IFNγ. Re-differentiation of epithelial cells was achieved by growing the cells under non-permissive conditions (37 or 39 °C and the absence of IFNγ). Taking this experimental approach using slight modifications of Whitehead et al. we successfully transformed Immortomouse^®^ derived CPECs by growing the cells at 33 °C and the addition of IFNγ as reflected by their high proliferation rate over several passages which could not be observed under non-permissive conditions. However, irrespective of the passage tested subsequent withdrawal of IFNγ and culture of the Immorto^®^CPEC lines at 37° or 39° failed to induce epithelial re-differentiation of the cells. Rather, we observed in all our Immorto^®^CPEC lines continuous expression of the mesenchymal markers vimentin and N-cadherin accompanied by the lack of re-expression of cytokeratin and E-cadherin. This suggests that immortalization of the Immorto^®^CPECs induced irreversible and thus SV40 large T independent epithelial to mesenchymal transition (EMT). Spontaneous induction of temperature-sensitive growth phenotypes of mouse embryonal fibroblasts, independent of a temperature-sensitive immortalizing gene, have been observed before [52]. Taken together our observations suggest that the Immortomouse^®^ is not well suited to establish CPEC lines that can be used to model the BCSFB in vitro.

We therefore next analyzed the mouse CP carcinoma cell line ECPC4, which was established from a CP carcinoma that had developed in transgenic mice expressing the SV40 large T antigen [34]. ECPC4 have been shown to exhibit a flattened, polygonal morphology and to maintain characteristics of CPECs such as expression of transthyretin (TTR) and α_2_-macroglobulin when originally characterized in passages 13–15. ECPC4 cells have subsequently been used to study the activation of the kallikrein-kinin system or expression of inducible nitric oxide synthase (iNOS) in the CP in innate immunity responses [53, 54] using unknown passages of the cells. In the present study, we found that the commercially available ECPC4 from passage 37 and higher, despite continued expression of TTR, lacked contact inhibition and did not express CPEC specific molecules such as cytokeratin or E-cadherin. Rather, all ECPC4 cells stained positive for vimentin and failed to establish CPEC specific junctional complexes. These observations suggested a significant stage of de-differentiation of ECPC4, which was further supported by our observation that ECPC4 failed to establish barrier properties such as high TEER and low permeability for small soluble tracers. Thus, ECPC4 are not suitable as in vitro model for the mouse BCSFB to study immune functions including the migration of T cells across this barrier.

In depth characterization of the primary mouse CPEC cultures in the present study demonstrated this approach to be a valid in vitro model to study immune functions of the mouse BCSFB. Primary mouse CPECs were isolated and grown at high purity and formed contact-inhibited monolayers. CPECs maintained expression of CPEC specific molecules and developed mature junctional complexes. Primary mouse CPEC established a tight barrier with high trans-epithelial electrical resistance (TEER) values comparable to the TEER measured across primary CPEC cultures from different species [16, 18–20, 55, 56] and across the BCSFB in vivo in cats [57]. Barrier characteristics of our pmCPEC cultures were further confirmed by the establishment of a permeability barrier for small soluble tracers such as 3 kDa dextran and Lucifer Yellow reaching Pe values that are comparable to the Pe values measured for the small tracer [^14^C]Sucrose across the BCSFB rat in vitro model [56, 58]. Furthermore, the permeability for 3 kDa dextran across the pmCPECs was more than fivefold lower than the permeability measured across the primary mouse brain microvascular endothelial cells pmBMECs assessed in our laboratory [38] and the permeability for Lucifer Yellow was more than twofold lower than across the pmBMECs [59] and threefold lower than across the novel human BBB model derived from hematopoietic stem cells [40].

Taken together cultured pmCPEC maintain important barrier characteristics of CPECs in vivo and are thus a suitable model for the mouse BCSFB in vitro.

To study T cell migration across the BCSFB in vitro, the pmCPEC cultures were adapted to grow on an inverted porous filter membranes as previously described in [37]. Interestingly, barrier characteristics of pmCPECs grown on the inverted side of the filter were significantly higher than those of pmCPECs grown on the upper side of the filter. As we failed to see any visible difference in morphology or in immunostaining of the pmCPEC monolayers, we cannot explain this difference at this time. We modelled the migration of encephalitogenic T cells across the non-stimulated and stimulated BCSFB in vitro and found that in the absence of inflammatory stimuli T cells migrated across the pmCPEC monolayer from the basolateral to the apical site. Addition of pro-inflammatory cytokines doubled the number of T cells crossing the pmCPEC monolayer in the same time—comparable to T cell diapedesis observed across a BBB in vitro model, respectively [60].

Taken together in the present study, we have successfully established an inverted in vitro filter model of the mouse BCSFB with barrier characteristics resembling that of the BCSFB in vivo. We provide proof of principle that this model is suited to study T cell migration from the blood to the CSF side across the BCSFB. This model of the mouse BCSFB can be used to investigate whether different immune cell subsets can cross the BCSFB and enable the cellular and molecular mechanism involved to be defined.

## Conclusions

A suitable tool for reproducible high throughput investigations of the molecular and cellular mechanisms that mediate the migration of specific immune cells into the CNS via the mouse BCSFB is missing to date. Here we show that cell line models derived from the Immortomouse^®^ and from the commercially available ECPC4 are not suited for this purpose. Rather pmCPECs growing on inverted Transwell filters were defined as a reliable in vitro model for experiments mimicking the T cell migration from the blood vessel side to the apical side facing the CSF. Moreover, our adapted ‘inverted’ BCSFB model is suitable for addressing not only the immune cell subset extravasation but also the passing of substances and pathogens, taking into account the correct orientation of the polarized cells as it occurs under physiological conditions within the brain.

## Abbreviations

AJ: adherence junction
APC: antigen presenting cell
Ara-C: cytosine arabinoside
BBB: blood–brain barrier
BCSFB: blood–cerebrospinal fluid barrier
CAM: cell adhesion molecule
CD: cluster of differentiation
CK: cytokeratin
CNS: central nervous system
CSF: cerebrospinal fluid
CP: choroid plexus
CS: calf serum
CPEC: choroid plexus epithelial cell
Da: dalton
DC: dendritic cell
DMEM: Dulbecco´s modified eagle medium
EAE: experimental autoimmune encephalomyelitis
E-Cad: epithelial cadherin
EGF: epidermal growth factor
EMT: epithelial to mesenchymal transition
F-actin: filamentous actin
FBS: fetal bovine serum
ICAM: intercellular adhesion molecule
IF: immunofluorescence
Ig: immunoglobulin
IGF: insulin like growth factor
INF-γ: interferon gamma
JAM: junctional adhesion molecule
LFA-1: leukocyte function associated antigen-1
LY: lucifer yellow
mAb: monoclonal antibody
MAdCAM: 1 mucosal vascular addressin cell adhesion molecule
MAM: migration assay medium
MCAM: melanoma cell adhesion molecule
MHC: major histocompatibility complex
MMP: matrix metalloprotease
MS: multiple sclerosis
OSP: oligodendrocyte specific protein
PBS: phosphate buffered saline
Pe: permeability coefficient
pmCPECs: primary mouse choroid plexus epithelial cells
PSGL-1: P-selectin glycoprotein ligand—1
SCI: spinal cord injury
SV: simian virus
TEER: transepithelial electrical resistance
TJ: tight junction
TNF-α: tumour necrosis factor alpha
ts: temperature sensitive
TTR: transthyretin
VCAM: vascular cell adhesion molecule
VLA-4: very late antigen-4
wt: wildtype
ZO: zonula occludens
